# Combined moderate and high intensity exercise with dietary restriction improves cardiac autonomic function associated with a reduction in central and systemic arterial stiffness in obese adults: a clinical trial

**DOI:** 10.7717/peerj.3900

**Published:** 2017-10-05

**Authors:** Min Hu, Shen Wang, Dan Wang, Qinhao Lai, Xiaoying Chen, Shiwei Duan, Mengke Zhao, Junhao Huang

**Affiliations:** 1 Guangdong Provincial Key Laboratory of Sports and Health Promotion, Scientific Research Center, Guangzhou Sport University, Guangzhou, China; 2 Department of Sports and Health, Guangzhou Sport University, Guangzhou, China; 3 School of Kinesiology, Shanghai University of Sport, Shanghai, China; 4 Medical Genetics Center, School of Medicine, Ningbo University, Ningbo, China

**Keywords:** Autonomic function, Arterial stiffness, Exercise, Obesity, Diet

## Abstract

**Objective:**

The present study aimed to assess the effects of exercise with dietary restriction on cardiac autonomic activity, arterial stiffness, and cardiovascular biomarkers in obese individuals.

**Methods:**

Seventeen obese adults completed an 8-week exercise and dietary program. Anthropometry, body composition, and multiple biochemical markers were measured. We used carotid-femoral pulse wave velocity (cfPWV), brachial-ankle pulse wave velocity (baPWV), central blood pressure, and augmentation index (AIx) to assess arterial stiffness. To determine cardiac autonomic activity, heart rate variability (HRV) was analyzed by standard deviation of normal-to-normal intervals (SDNN), square root of the mean squared differences of successive normal-to-normal intervals (RMSSD), total power (TF), low-frequency power in normalized units (LFnu), high-frequency power in normalized units (HFnu), and low-frequency power/high-frequency power (LF/HF).

**Results:**

Following the exercise and diet intervention, obese subjects had significant reductions in body weight, body mass index, body fat percentage, brachial systolic blood pressure, and resting heart rate, and they had shown improvements in blood chemistry markers such as lipid profiles, insulin, and high-sensitivity C-reactive protein. There was a significant reduction in both cfPWV and baPWV following the intervention when compared to baseline levels. Moreover, the AIx and aortic systolic blood pressure were significantly reduced after the intervention. The diet and exercise intervention significantly increased cardiac autonomic modulation (determined by improved SDNN, RMSSD, TP LF, HF, and LF/HF), which was partly due to changes in heart rate, insulin resistance, and the inflammatory pattern. Furthermore, we observed a correlation between enhanced cardiac autonomic modulation (LF/HF) and decreased arterial stiffness, as measured by central cfPWV and systemic baPWV.

**Discussion:**

An 8-week combined intervention of diet and exercise is effective in improving cardiac autonomic function in obese adults, with an associated decrease in central and systemic arterial stiffness.

## Introduction

Abnormalities in arterial function contribute to an increased risk of cardiovascular events in obese patients (Ortega, Lavie & Blair, 2016). Previous studies have established that obesity is positively correlated with arterial stiffness, which is a key characteristic of atherosclerosis (Acree et al., 2007; Van Guilder et al., 2006). Pulse wave velocity (PWV) has been recognized as the most largely employed method to assess arterial stiffness (Cardoso & Salles, 2016). Aortic arterial stiffness is evaluated by carotid-femoral PWV (cfPWV), and systemic arterial stiffness is determined by brachial-ankle PWV (baPWV). These markers are considered to be simple and reliable indicators of conditions of central and systemic arterial function.

A strong link has been identified between obesity and autonomic dysfunction (Piestrzeniewicz et al., 2008). The autonomic nervous system plays a vital role in regulating heart rate and vascular tone (Jaiswal et al., 2013). Thus, impaired autonomic function may contribute to increased arterial stiffness in obese individuals (Figueroa et al., 2012). Furthermore, recent studies have suggested an inverse correlation between autonomic activity and arterial stiffness in patients with type 1 diabetes (Jaiswal et al., 2013) and systemic sclerosis (Gigante et al., 2014). Although the evidence linking autonomic function and arterial stiffness in healthy and diseased states has been shown, it is still largely unexplored in this area under physiological and pathological conditions (Amiya, Watanabe & Komuro, 2014). Therefore, the elucidation of the association between these cardiovascular biomarkers may provide a new effective therapeutic strategy against cardiovascular disease.

Previous clinical trials have reported that exercise and diet intervention improve the obesity-associated cardiovascular biomarkers of body composition, blood pressure, dyslipidemia, insulin resistance, and inflammation. Additionally, recent studies have demonstrated that exercise or dietary modification may improve cardiac autonomic function (Felber Dietrich et al., 2008; Lind & Andren, 2002) and arterial stiffness (Goldberg et al., 2009; Maeda et al., 2015) in obese subjects. However, there have been few studies focusing on the specific relationship between autonomic function and arterial stiffness under lifestyle modifications of exercise and diet.

Thus, the present study examined the relationship between autonomic function and arterial stiffness in people with obesity after an 8-week program of combined exercise and dietary restriction. We hypothesized that exercise and diet intervention would improve autonomic function associated with decreased arterial stiffness.

## Materials and Methods

The trial forms part of a larger study with lifestyle interventions on obese adults (Effects of exercise training and diet restriction on cardiovascular function in obese population, ISRCTN83594346). Results regarding the relationship between circulating irisin concentrations and endothelial progenitor cell levels in an obese population after exercise and dietary intervention have been reported in an earlier publication (Huang et al., 2017a). In the present paper, we focus solely on the relationship between autonomic function and arterial stiffness under lifestyle modifications of exercise and diet. This study was conducted following the ethical guidelines of the Declaration of Helsinki, and the study was approved by the Ethics Committee of Guangzhou Sport University (approval No. GSU20160012). All participants provided written informed consent prior to the measurements. The study protocol and supporting TREND checklist were provided as Supplemental Information; see S1 Protocol and S1 Checklist.

### Participants

**Figure 1:**
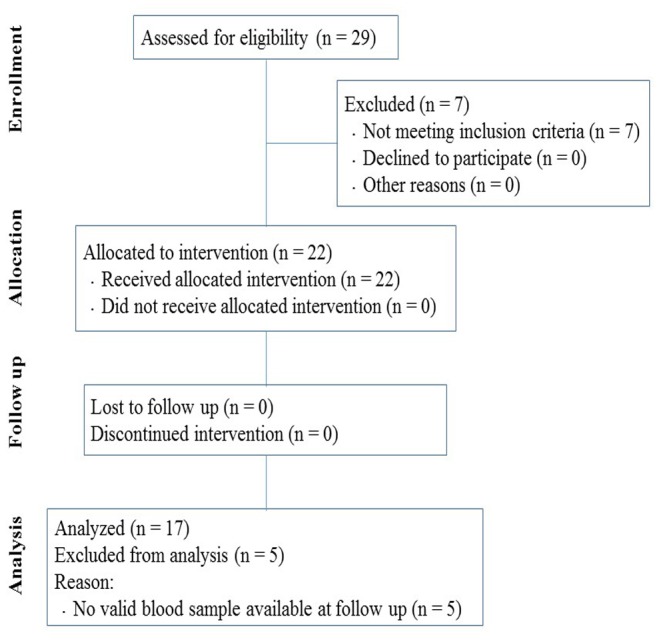
Flow diagram of participants through the research program.

Obese adults between the ages of 18 and 40 years (body mass index (BMI) ≥ 30 kg/m^2^) were recruited from a weight loss camp after a physical examination. Those with diabetes, unstable angina pectoris, cardiomyopathy, severe lung diseases, or renal failure were excluded. Of the twenty-nine enrolled participants, twenty-two met the criteria for inclusion and were selected for the training program. Seventeen participants (M/F, 11/6) who completed the program and provided valid measurements at both baseline and follow-up were included in the analysis (TREND flow diagram, Fig. 1).

### Exercise and diet intervention

Details for the diet and exercise protocol have been previously described (Huang et al., 2017a). Briefly, subjects were provided with calorie-restricted diets of 1,300–2,200 kcal/day based on their weight. Energy intakes were calculated using the Harris-Benedict equation (Yassine et al., 2009). The targeted daily caloric intake deficit was 500 kcal/day. The diet consisted of balanced proportions of 60% carbohydrate, 20% protein, and 20% fat, and calories were distributed as 30%, 40%, and 30% at breakfast, lunch, and dinner, respectively. The dietitians prepared and supervised all meals. Subjects performed a series of endurance exercises including bicycling, walking, running, dancing, and ball games for 5 h/day. The endurance exercises involved moderate and high intensity training. The intensity of moderate-intensity exercise was set at 70–85% of the subject’s maximum heart rate (HRmax), which was calculated with the formula of 208 − (0.7 × *age*). The high-intensity exercise (∼90% of HRmax) was alternated with low-intensity exercise (∼60% of HRmax) during training. Subjects were also supplemented with strength training. After measuring maximal strength, strength exercise was performed at 40–50% maximal strength for 2–3 sets of 12–15 repetitions with 2–3 min rest between each set. Exercise interventions were performed 6 days/week for 8 weeks. A daily training log assessed the subject’s compliance was recorded. The exercise training was designed to lead to an energy expenditure of 1,500–2,500 kcal/day and was supervised by qualified trainers. Measurements were performed before the training and 24 h after last session of the 8-week exercise and diet program.

### Body composition and biochemical factors

Height and weight were determined according to previously described method (Hu et al., 2017), and BMI was calculated (kg/m^2^). Body composition was assessed by a body composition analyzer (Inbody 370; Biospace, Seoul, Korea). Fasting blood samples were taken in the early morning for the assessment of total cholesterol, triglycerides, high-density lipoprotein cholesterol (HDL-c), low-density lipoprotein cholesterol (LDL-c), glucose, and insulin. Insulin resistance was evaluated using the Homeostasis Model of Assessment of Insulin Resistance (HOMA-IR) and was calculated as the [fasting insulin (µU/ml) × fasting glucose (mmol/L)]/22.5. Concentration of high-sensitivity C-reactive protein (hsCRP) in serum was analyzed using an ELISA Kit (Cusabio; Biotech. Co., LTD, Wuhan, China) in accordance with the manufacturer’s instruction.

### Resting heart rate and brachial blood pressure

Resting heart rate (HR) and brachial systolic/diastolic blood pressure (SBP/DBP) were measured three times by a sphygmomanometer. The participants were requested to rest for at least 10 min, and the average of three measurements was taken.

### Heart rate variability (HRV)

HRV was measured using the SphygmoCor device (AtCor Medical, Sydney, Australia), following previously described protocols (Figueroa et al., 2012; Jaiswal et al., 2013). As for the time domain analysis, we determined the standard deviation (SD) of normal-to-normal (NN) intervals (SDNN) and square root of the mean squared differences of successive NN intervals (RMSSD). For frequency domain analysis, total power (TP) in the frequency (0–0.40 Hz) was divided into low-frequency power (LF: 0.04–0.15 Hz, modulated mainly by sympathetic system) and high-frequency power (HF: 0.15–0.40 Hz, modulated by parasympathetic system). Normalized units (nu) were calculated by dividing the power of LF or HF by (LF + HF). LF/HF ratio reflects the balance between sympathetic and parasympathetic activity (sympathovagal balance).

### Pulse wave velocity

cfPWV was measured according to previously described technique (Laurent et al., 2006; Logan & Barksdale, 2013). The transit time of the pulse wave from the left ventricle to the carotid artery (t1) and from the left ventricle to the femoral artery (t2) were measured by a pressure sensitive transducer (tonometer). The distance from the suprasternal notch to the carotid artery (d-carotid) and from the suprasternal notch to the femoral artery (d-femoral) were calculated using a measuring tape. The cfPWV was calculated as the difference in distance between d-femoral and d-carotid divided by the time difference between t1 and t2. Additionally, baPWV and cfPWV were also calculated with an oscillometric device (boso ABI-system 100; BOSCH & SOHN, Germany).

### Pulse wave analysis

Aortic pressure waveforms were derived using a generalized validated transfer function (SphygmoCor device) (Figueroa, Wong & Kalfon, 2014; Laurent et al., 2006; Sanchez-Gonzalez et al., 2012). Briefly, the aortic pressure wave is composed of a forward wave, created by ventricular contraction, and a reflected wave that returns to the aorta from the periphery. Augmentation pressure is the difference between the second and first systolic peaks (Figueroa, Wong & Kalfon, 2014). The augmentation index (AIx) was defined as the augmentation pressure expressed as a percentage of the aortic pulse pressure (Sanchez-Gonzalez et al., 2012). Since AIx is influenced by heart rate, it is normalized to a standard heart rate of 75 beats/min (AIx75).

### Statistical analysis

Analyses were performed using SPSS 16.0 (SPSS Inc., Chicago, IL, USA). Paired-sample t-tests were used to compare the data obtained before and after the intervention. Pearson correlation analysis was performed to measure the association between two variables. Data are reported as mean ± SD. A *P*-value < 0.05 is used to indicate statistical significance.

## Results

### Effects of exercise and diet intervention on anthropometric and metabolic parameters

The general outcomes (descriptive characteristics, whole body composition, and metabolic parameters) measured before and after the 8-week training program of obese participants have been previously described in detail (Huang et al., 2017a). As shown in Table 1, in response to the exercise and diet intervention, obese participants (22.1 ± 2.8 years) had significant reductions in body weight, BMI, body fat mass, and body fat percentage (all *P* < 0.001). Obese subjects also had significantly lower concentrations of serum cholesterol (*P* < 0.05), triglycerides (*P* < 0.01), LDL-c (*P* < 0.05), insulin (*P* < 0.01), and HOMA-IR (*P* < 0.01) following intervention. Whereas, no significant changes in levels of blood glucose and HDL-c were found (Table 1). Moreover, the intervention markedly reduced inflammation, as shown by a decline in hsCRP levels (*P* < 0.01; Table 1).

**Table 1:** The anthropometric and metabolic parameters of subjects before and after 8-week combined exercise and diet intervention.

Parameters	Before	After
Age (yr)	22.1 ± 2.8	–
Gender (*n*) (M/F)	17 (11/6)	–
Height (cm)	174.8 ± 1.85	–
Body weight (kg)	116.6 ± 24.6	103.4 ± 22.4^∗∗∗^
BMI (kg/m^2^)	37.8 ± 5.0	33.5 ± 4.4^∗∗∗^
Body fat (kg)	47.4 ± 12.7	38.2 ± 12.2^∗∗∗^
Body fat (%)	40.9 ± 4.6	36.6 ± 5.9^∗∗∗^
Cholesterol (mmol/l)	5.35 ± 0.92	4.74 ± 1.34^∗^
Triglycerides (mmol/l)	2.15 ± 1.08	1.32 ± 0.88^∗∗^
HDL-c (mmol/l)	0.99 ± 0.14	0.99 ± 0.22
LDL-c (mmol/l)	3.44 ± 0.75	2.99 ± 0.95^∗^
Fasting blood glucose (mg/dl)	5.61 ± 0.56	5.39 ± 0.57
Fasting insulin (pmol/l)	200.8 ± 115.8	116.7 ± 81.2^∗∗^
HOMA-IR	7.34 ± 4.9	4.08 ± 2.9^∗∗^
hsCRP (µg/ml)	2.49 ± 1.71	1.36 ± 1.61^∗∗^

**Notes.**

BMI body mass index HDL-c high-density lipoprotein cholesterol LDL-c low-density lipoprotein cholesterol HOMA-IR Homeostasis Model of Assessment of Insulin Resistance hsCRP high-sensitivity C-reactive protein

Values are presented as mean ± SD. ^∗^ *P* < 0.05, ^∗∗^ *P* < 0.01, ^∗∗∗^*P* < 0.001 vs. Before.

### Effects of exercise and diet intervention on cardiovascular function

Using the boso ABI-system 100 device, we observed a reduction after intervention in the right baPWV relative to baseline (9.78 ± 1.25 m/s vs. 9.32 ± 0.88 m/s, *P* < 0.05). However, no significant difference was observed in the left baPWV (Table 2). Additionally, we found a reduction in cfPWV using both the boso device (5.91 ± 0.98 m/s vs. 5.58 ± 0.59 m/s, *P* < 0.05) and SphygmoCor device (6.21 ± 1.55 m/s vs. 5.44 ± 1.30 m/s, *P* < 0.05). Moreover, obese individuals showed significant reductions in augmentation pressure (*P* < 0.05), AIx75 (*P* < 0.01), and resting heart rate (*P* < 0.001) after the intervention (Table 2). There was a significant decrease in brachial SBP (*P* < 0.001), brachial pulse pressure (*P* < 0.05), aortic SBP (*P* < 0.001), and aortic pulse pressure (*P* < 0.05) following exercise and diet intervention (Table 2). However, there were no changes in brachial and aortic DBP.

**Table 2:** The cardiovascular function parameters of subjects before and after 8-week combined exercise and diet intervention.

Parameters	Before	After
Left baPWV (m/s)^a^	9.59 ± 1.28	9.45 ± 0.81
Right baPWV (m/s)^a^	9.78 ± 1.25	9.32 ±0.88^∗^
cfPWV (m/s)^a^	5.91 ± 0.98	5.58 ±0.59^∗^
cfPWV (m/s)^b^	6.21 ± 1.55	5.44 ±1.30^∗^
Augmentation pressure (mmHg)^b^	2.50 ± 3.16	1.31 ±2.41^∗^
AIx75 (%)^b^	7.50 ± 12.09	−0.25 ±13.49^∗∗^
HR (bpm)	74 ± 9	61 ±9^∗∗∗^
bSBP (mmHg)	127.8 ± 11.8	115.3 ±7.4^∗∗∗^
bDBP (mmHg)	85.2 ± 9.4	79.9 ± 10.8
bPP (mmHg)	42.6 ± 6.40	35.4 ±9.14^∗^
aSBP (mmHg)^b^	112.81 ± 10.51	102.94 ±8.67^∗∗∗^
aDBP (mmHg)^b^	86.50 ± 10.11	80.75 ± 11.17
aPP (mmHg)^b^	26.50 ± 5.29	22.19 ±5.18^∗^

**Notes.**

baPWV brachial-ankle pulse wave velocity cfPWV carotid-femoral pulse wave velocity AIx75 augmentation index standardized to a heart rate of 75/min HR heart rate bSBP brachial systolic blood pressure bDBP brachial diastolic blood pressure bPP brachial pulse pressure aSBP aortic systolic blood pressure aDBP aortic diastolic blood pressure aPP aortic pulse pressure

Values are presented as mean ± SD. ^∗^ *P* < 0.05, ^∗∗^ *P* < 0.01, ^∗∗∗^*P* < 0.001 vs. Before.

a Parameters were measured by boso ABI-system 100.

b Parameters were measured by SphygmoCor device.

### Effects of exercise and diet intervention on cardiac autonomic function

When focusing on the HRV indices of obese individuals at the baseline and at the end of 8 weeks of training program (Table 3), we found a significant augmentation in levels of SDNN (47.74 ± 26.41 ms vs. 65.19 ± 24.52 ms, *P* < 0.01) and RMSSD (39.43 ± 36.67 ms vs. 59.29 ± 37.61 ms, *P* < 0.05) using time domain assessment. Moreover, we detected a significant improvement in frequency domain parameters (i.e., TP, LFnu, HFnu, and LF/HF; all *P* < 0.05).

**Table 3:** The heart rate variability indices of subjects before and after 8-week combined exercise and diet intervention.

Parameters	Before	After
SDNN (ms)	47.74 ± 26.41	65.19 ±24.52^∗∗^
RMSSD (ms)	39.43 ± 36.67	59.29 ±37.61^∗^
TP (ms^2^)	1672 ± 2467	3155 ±3298^∗^
LFnu	55.45 ± 24.56	44.60 ±17.49^∗^
HFnu	44.55 ± 24.56	55.40 ±17.49^∗^
LF/HF	2.34 ± 2.52	1.06 ±0.92^∗^

**Notes.**

SDNN standard deviation of normal-to-normal intervals RMSSD square root of the mean squared differences of successive normal-to-normal intervals TP total power LFnu low-frequency power in normalized units HFnu high-frequency power in normalized units LF/HF low-frequency power/high-frequency power

Values are presented as mean ± SD. ^∗^ *P* < 0.05, ^∗∗^ *P* < 0.01 vs. Before.

### Correlations

As shown in Table 4, correlation analysis showed that the change in cfPWV was positively correlated with the change in LFnu (*r* = 0.516, *P* < 0.05); however, changes in cfPWV were negatively correlated with changes in HFnu (*r* =  − 0.516, *P* < 0.05). Both of the changes in cfPWV and right baPWV were positively associated with changes in LF/HF (*r* = 0.601, *P* < 0.05 and *r* = 0.498, *P* < 0.05, respectively). This suggests there is a significant correlation between reduced arterial stiffness and enhanced cardiac autonomic modulation following an exercise and diet intervention. The change in HR demonstrated a significant negative correlation with changes in HFnu (*r* =  − 0.587, *P* < 0.05), SDNN (*r* =  − 0.618, *P* < 0.05), and RMSSD (*r* =  − 0.643, *P* < 0.05). In contrast, there was a positive correlation between changes in HR and LFnu (*r* = 0.587, *P* < 0.05). We also found that changes in insulin resistance was positively correlated with changes in LFnu (*r* = 0.490, *P* < 0.05) and negatively correlated with changes in HFnu (*r* =  − 0.490, *P* < 0.05). Moreover, we observed a negative correlation between changes in hsCRP and both changes in TP and RMSSD (*r* =  − 0.777, *P* < 0.01 and *r* =  − 0.738, *P* < 0.01, respectively; Table 4).

**Table 4:** Associations of changes in heart rate variability indices with changes in pulse wave velocity, heart rate, insulin sensitivity and inflammatory pattern after 8-week combined exercise and diet intervention.

	cfPWV	Right baPWV	HR	HOMA-IR	hsCRP
TP	−0.101	−0.083	−0.286	−0.181	−0.777^**^
LFnu	0.516^*^	0.327	0.587^*^	0.490^*^	0.290
HFnu	−0.516^*^	−0.327	−0.587^*^	−0.490^*^	−0.290
LF/HF	0.601^*^	0.498^*^	0.307	0.099	−0.063
SDNN	−0.496	−0.269	−0.618^*^	−0.439	−0.149
RMSSD	−0.366	−0.125	−0.643^*^	−0.392	−0.738^**^

**Notes.**

TP total power LFnu low frequency in normalized units HF high frequency in normalized units LF/HF low frequency/high frequency SDNN standard deviation of normal-to-normal intervals RMSSD square root of the mean squared differences of successive normal-to-normal intervals cfPWV carotid-femoral pulse wave velocity baPWV brachial-ankle pulse wave velocity HR heart rate HOMA-IR Homeostasis Model of Assessment of Insulin Resistance hsCRP high-sensitivity C-reactive protein

* *P* < 0.05.

** *P* < 0.01.

## Discussion

The main findings of the current study were an observed improvement in cardiac autonomic activity by enhancing parasympathetic modulation and decreasing sympathetic modulation, correlated with a reduction in central and systemic arterial stiffness in people with obesity after an 8-week program consisting of exercise and dietary restriction.

Obesity is frequently accompanied by atherosclerotic plaque formation (Tounian et al., 2001; Woo et al., 2004). Early prognosis of arterial stiffness can prevent obese people from suffering vascular disease. In the current study, we used two methods to measure cfPWV, and both methods detected a significant improvement in cfPWV after the intervention (Table 2). The levels of baPWV may represent the dilation of the aorta and the lower-limb artery (Munakata et al., 2012). High baPWV is closely correlated with weak arterial dilation, high stiffness, and poor elasticity. Evaluation of baPWV has become a new tool for measuring arterial stiffness because of its convenience and easy operation. In the current study, we found a significant reduction in right baPWV rather than left baPWV after the intervention, which suggests a location-specific effect of diet and exercise intervention on arterial stiffness. Moreover, we found that the change in baPWV was highly correlated with the change in cfPWV (*r* = 0.929, *P* < 0.001), and this is in accordance with what has been elucidated previously (Yamashina et al., 2002). Therefore, it indicates that baPWV is a good method for use in studies measuring arterial stiffness because it has high reproducibility.

HRV is frequently decreased in patients with obesity or diabetes and is associated with a poor cardiovascular prognosis (La Rovere et al., 2001). Therefore, it is important to improve HRV in these patients with therapeutic approaches. To assess the overall autonomic activity, we used SDNN (Salamin et al., 2013), and we found a significant increase in SDNN with enhanced overall parasympathetic activity demonstrated by RMSSD. This is consistent with a previous study in which a weight reduction program for children with obesity induced a change in autonomic activity toward parasympathetic dominance (Mazurak et al., 2016). Furthermore, our study revealed that TP, LFnu, HFnu, and LF/HF were significantly improved after the intervention. This suggests improved autonomic regulation by ways of increased parasympathetic modulation and decreased sympathetic modulation. A possible explanation for the HRV variations found in our study is that the exercise and diet intervention improved HR, insulin resistance, and inflammatory pattern, and these outcomes are important to the regulation of cardiac autonomic activity (Giallauria et al., 2008; Nakao et al., 2004). Indeed, we observed a significant negative correlation between augmented HRV and the changes in these cardiovascular biomarkers (Table 4). These results suggest the effects of an 8-week program of exercise and dietary restriction on cardiac autonomic modulation were partly due to the changes in these biomarkers.

A novel finding of our study was that a decreased sympathovagal balance (as assessed by LF/HF) was correlated with a reduction in PWV (i.e., cfPWV and baPWV) after an exercise and diet intervention. To the best of our knowledge, this is the first study to report a significant association between improved autonomic function and reduced vascular stiffness in both central and systemic vascular beds among obese adults undergoing lifestyle modifications of exercise and diet intervention. In accordance with our results, a previous study showed that 6 weeks of whole-body vibration training in young overweight and obese women reduced both baPWV and sympathovagal balance (Figueroa et al., 2012). The mechanisms underlying the association between changes in cardiac autonomic function and arterial stiffness after life modifications need to be elucidated in future studies.

A limitation of our current study is the lack of a control group during the training program of the intervention. Since the camp was located in a remote district of Huizhou city, China, and subjects enrolled in the program came from various locations across the country and had an average BMI over 35 kg/m^2^ (37.8 ± 5.0 kg/m^2^), the selection of an appropriate control group was restricted (Gately et al., 2000; Huang et al., 2017a). Furthermore, we were limited by a small sample size. In future, we will also be interested in probing potential changes in central nervous system following exercise with diet intervention, such as using motor evoked potentials and its dynamic plasticity (Huang et al., 2017b; Shen et al., 2017). These limitations are important and should be addressed in the future.

In conclusion, the present study found a correlation between an enhancement of cardiac autonomic function and a reduction in central and systemic arterial stiffness in obese adults after 8 weeks of exercise training with dietary restriction.

##  Supplemental Information

10.7717/peerj.3900/supp-1 Data S1 S1 Raw data

10.7717/peerj.3900/supp-2 Supplemental Information 1 S1 Checklist

10.7717/peerj.3900/supp-3 Supplemental Information 2 S1 Study Protocol

10.7717/peerj.3900/supp-4 Supplemental Information 3 S2 Study protocol in original language (Chinese)

## Additional Information and Declarations

### Data Availability

The following information was supplied regarding data availability: The raw data is included as a Supplemental File.

### Clinical Trial Registration

The following information was supplied regarding Clinical Trial registration:
ISRCTN83594346.
